# Utilizing CRISPR-Cas in Tropical Crop Improvement: A Decision Process for Fitting Genome Engineering to Your Species

**DOI:** 10.3389/fgene.2021.786140

**Published:** 2021-11-12

**Authors:** Kathleen A. Joo, Michael G. Muszynski, Michael B. Kantar, Ming-Li Wang, Xiaoling He, Angel R. Del Valle Echevarria

**Affiliations:** ^1^ Department of Tropical Plant and Soil Sciences, University of Hawaii at Manoa, Honolulu, HI, United States; ^2^ Hawaii Agriculture Research Center, Waipahu, HI, United States

**Keywords:** non-commodity, science democratization, orphan crop, genome engineering, CRISPR-cas, tropical crop

## Abstract

Adopting modern gene-editing technologies for trait improvement in agriculture requires important workflow developments, yet these developments are not often discussed. Using tropical crop systems as a case study, we describe a workflow broken down into discrete processes with specific steps and decision points that allow for the practical application of the CRISPR-Cas gene editing platform in a crop of interest. While we present the steps of developing genome-edited plants as sequential, in practice parts can be done in parallel, which are discussed in this perspective. The main processes include 1) understanding the genetic basis of the trait along with having the crop’s genome sequence, 2) testing and optimization of the editing reagents, development of efficient 3) tissue culture and 4) transformation methods, and 5) screening methods to identify edited events with commercial potential. Our goal in this perspective is to help any lab that wishes to implement this powerful, easy-to-use tool in their pipeline, thus aiming to democratize the technology.

## Introduction

Since its proposal as a eukaryotic gene-editing tool (Jinek et al., 2012), the Clustered Regularly Interspaced Short Palindromic Repeats (CRISPR) and CRISPR-Associated protein (Cas) technology has been widely applied in microorganisms, animals, and plants to study gene function (Haque et al., 2018) due to its simplicity in design and straightforward execution. Numerous CRISPR-Cas system reviews explain its discovery and aspects to consider when using this powerful molecular tool, so we suggest reviewing Anzalone et al., 2020 for details on specifics, such as the diverse engineered Cas nucleases. For this perspective, the reader must know that the CRISPR-Cas tool is composed of a small guide RNA (sgRNA) complementary to a DNA target sequence and a Cas endonuclease (i.e., Cas9 and Cas12a). The Cas endonuclease recognizes a protospacer adjacent motif (PAM) sequence that is upstream [5′-TTTV-(22 nt of target sequence)-3′] for Cas12a or downstream [5′-(20 nt of target sequence)-NGG-3′] for Cas9 of the target sequence (Jinek et al., 2012; Zetsche et al., 2015). The Cas nuclease associates with the sgRNA to form a ribonucleoprotein (RNP) complex, which scans the genome for the PAM sequence and, by complementation, the DNA target sequence (Jinek et al., 2012; Zetsche et al., 2015). The RNP complex catalyzes a double-strand break (DSB) in the target DNA, triggering the cell’s error-prone DNA repair mechanism, which results in the creation of a mutation that may generate a desirable change in a trait of interest (Woo et al., 2015).

In agriculture, CRISPR-Cas technology has been used to introduce added-value traits (Tian et al., 2018; Kaur et al., 2020; Yoon et al., 2020) and to recapitulate domestication processes (Soyk et al., 2017, 2019; Lemmon et al., 2018). Based on these examples, it is clear that the application of the CRISPR-Cas tool, coupled with traditional breeding practices, has tremendous potential in alleviating threats to food security, production, and sustainability (Jung and Till, 2021). Numerous published studies state that the CRISPR-Cas tool can be used in any crop due to its simplicity, but there are key aspects to consider when applying this technology to specific types of crops.

**FIGURE 1:**
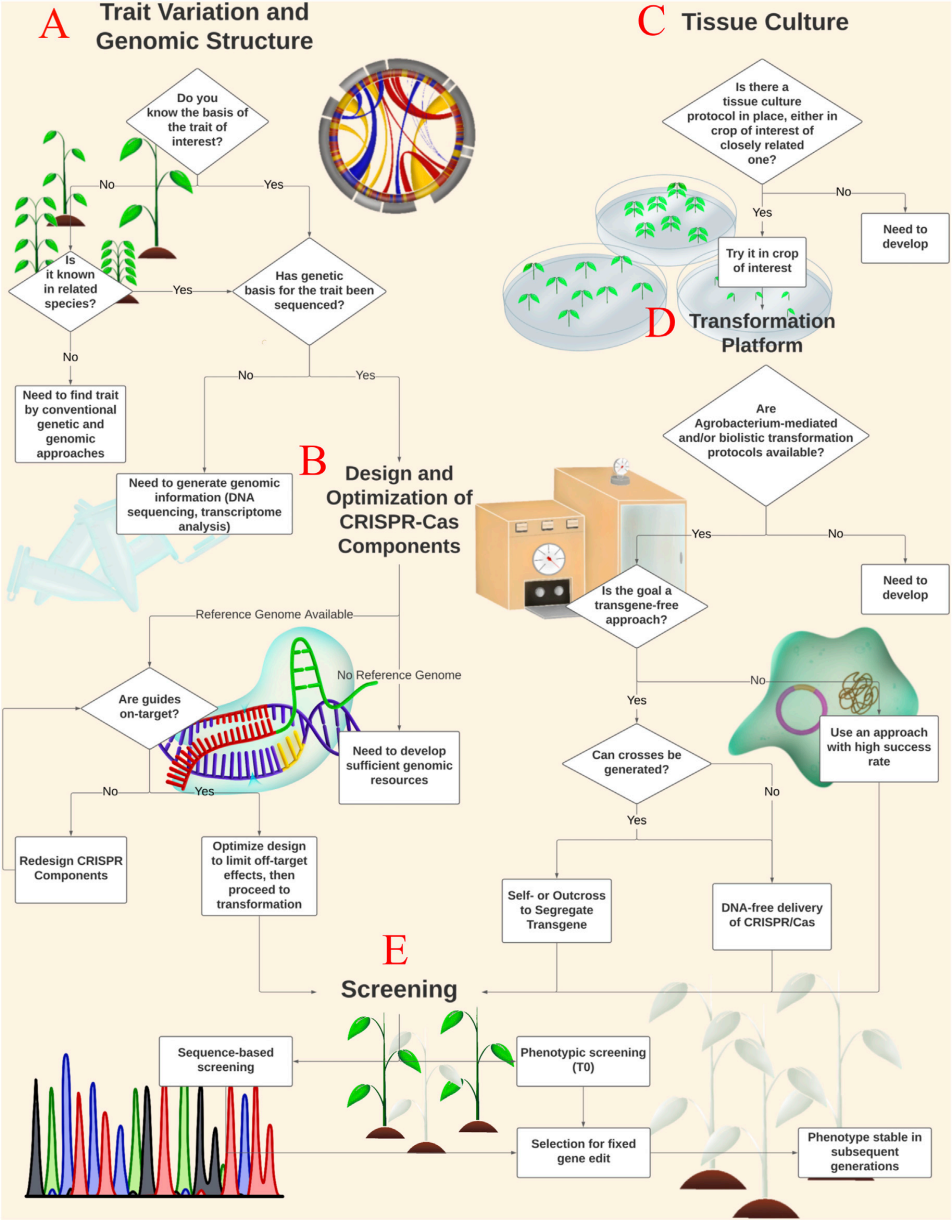
CRISPR-Cas Gene Editing Workflow. Here we present a schematic for implementing CRISPR-Cas gene editing technology in a crop of interest. Diamonds represent decision points, while boxes represent important processes. In order to develop a gene editing protocol using CRISPR-Cas technology, the genetic basis for the trait of interest must be identified and well described **(A)**. If genomic information is non-existent or limited for the varietal of interest, it must be extrapolated from existing data on similar genotypes or from related species. Adequate genomic information is essential for the design and optimization **of** CRISPR-Cas sgRNA, nuclease selection, and off-target analysis **(B)**. Identification of the genetic basis for a trait **(A)** and design of CRISPR-Cas components **(B)** constitute the genomic aspect of the workflow, and consideration for both can take place independently of tissue culture **(C)** and transformation **(D)**. We suggest testing various tissue culture protocols when they do not exist for the varietal of interest but do exist for a different genotype or related species **(C).** CRISPR-Cas reagents may be introduced to cultured cells in several ways including *Agrobacterium* ***-*** mediated transformation, transient expression from transfected plasmids, and biolistic transformation with RNP or RNA complexes. Selection of the transformation method will depend on the goals of the research. Following CRISPR-Cas design **(B)** and transformation **(D)**, explants should be screened, field tested, and propagated **(E)** to generate a phenotypically stable, CRISPR-Cas gene edited population. Screening for edited events **(E)** will involve sequencing for allelic differences and phenotypic selection. Depending on the goals of the research, marker-guided selection and resistance genes may also be used to screen for edited events.

Tropical crops present an appropriate case study where implementation of the CRISPR-Cas technology would significantly contribute to its improvement. However, a lack of genomic resources, complex tissue culture procedures, and reproductive compatibility constraints in many tropical crops make utilizing this robust tool challenging (Atkins and Voytas, 2020; Maher et al., 2020). Although promising advances in tropical crop improvement using CRISPR-Cas have recently been achieved, most focus on proof-of-concept studies (Odipio et al., 2017; Naim et al., 2018; Fan et al., 2020; Ntui et al., 2020; Eid et al., 2021; Syombua et al., 2021; Zhao et al., 2021) and few on value-added traits (Gomez et al., 2017; Bull et al., 2018; Fister et al., 2018; Mehta et al., 2019; Oz et al., 2021). In this perspective we outline critical points to consider when executing a gene-editing project in a tropical crop species (Figure 1). This perspective will outline key considerations at the main decision points throughout the gene editing workflow.

### Trait Variation and Genomic Structure

The foundation of any gene-editing project relies on understanding the genetic mechanism(s) underlying trait variation within the species of interest and identifying where to source specific varietal material (i.e., genebanks, elite lines). There are numerous international and regional institutions dedicated to conserving tropical collections (FAO, 2010). However, inherent properties of common tropical crops, such as recalcitrant seeds (Bourdeix et al., 2020), low seed number or viability (Batte et al., 2019; Kallow et al., 2020; Mertens et al., 2021), and laborious maintenance of vegetatively propagated crops (Balogun, 2009; Gaba and Singer, 2009; Panis et al., 2020), tend to limit collection size.

Plant breeding efforts in tropical crops often focus on domestication traits first, followed by value-added commercialization traits (Ramstein et al., 2019; Bernardo, 2020). Several challenges for efficient breeding in tropical crops include polyploidy, clonal propagation, and obligate outcrossing biology (Santantonio et al., 2020). Furthermore, lengthy growth cycles (Batugal and Bourdeix, 2005; Wickramasuriya and Dunwell, 2018) and unpredictable or asynchronous flowering (Amadi et al., 2012; Darkwa et al., 2020) in some tropical crops make breeding intractable. Tropical crops that have not become global commodities are often clonal, and therefore have traits associated with domestication syndrome that are not “fixed” (Denham et al., 2020). The lack of research on many of these crops also impacts the identification of genotypes with more desirable characteristics (Varshney et al., 2012). Outbreeding tropical crops tend to be highly heterozygous (Ceballos et al., 2004; Wickramasuriya and Dunwell, 2018; Darkwa et al., 2020), making inbred and double-haploid lines difficult to generate (Ceballos et al., 2004). The inability to create inbred lines, which are required for straightforward prediction of genetic gain and consistent improvement of breeding material (Bernardo, 2014; Cobb et al., 2019), limits the efficiency of conventional breeding in many tropical crops and often makes knowing the exact genetic basis of a trait difficult. However, genome engineering becomes a very enticing technology for crop improvement when the genetic basis has been identified, often in a tractable model species.

Critical to the development of a gene-editing workflow is understanding the genetic variation that underlies trait expression (Figure 1A). Genetic mapping is typically used to identify the relative location of genes associated with specific traits on a chromosome, though the process becomes more complex in polyploid organisms. To date, success in gene editing has focused on traits following qualitative, Mendelian inheritance [i.e., few, large effect gene(s)] since many domestication traits behave in this manner. For example, plant architecture (Li et al., 2016; Lemmon et al., 2018; Zhang et al., 2020), flowering time (Soyk et al., 2017), and some disease resistance traits (Macovei et al., 2018) have been studied in detail in model organisms which identified their genetic bases, making them attractive targets for similar modification in tropical crops. Therefore, the identity of the underlying genetic cause of the trait is key to being able to “introduce” such traits into the crop of interest.

Another important workflow component is an annotated reference genome, which allows for quick identification of target sequences and homologous sites for genome editing (Bortesi and Fischer, 2015). In cases where little to no genomic information is available, information should be extrapolated from existing genomic resources of closely related species, an approach Lemmon et al. (2018) used to target domestication traits in the orphan crop “groundcherry.” Though genome size and ploidy level often cause challenges in genome assembly, reference genomes do exist for several tropical crops (Ming et al., 2008; Argout et al., 2011; Prochnik et al., 2012; Xiao et al., 2017; Lantican et al., 2019; Sahu et al., 2020; Wang et al., 2020; Bally et al., 2021; Strijk et al., 2021). Pan-genomes, if available, offer a more robust view of a cultivar’s genetic variation since they consist of a core genome shared by all sequenced individuals, and reveal genetic variations that are present or absent in re-sequenced genomes (Kyriakidou et al., 2018; Marschall et al., 2018).

### Design and Optimization of CRISPR-Cas Components

Sequence information about the gene or region in the chromosome associated with the trait of interest is critical in designing the appropriate gRNA respective to the Cas endonuclease (Figure 1B). Having the genomic sequence allows for identification of the PAM sequence relevant to the Cas enzyme to be used (i.e., 5′-NGG-3′ for Cas9 (Jinek et al., 2012) or 5′-TTTV for Cas12a (Zetsche et al., 2015), where “N” is any nucleotide and “V” is A, G, or C) and subsequent gRNA design. Several software programs are available that screen for PAM sequences based on the Cas enzyme of interest and suggest guide designs that target the region with a quality score. If a reference genome is available, the software can also identify potential off-target sites (Brazelton et al., 2015; Doench et al., 2016). The guides can be synthesized in-house via *in vitro* transcription (Liang et al., 2017; 2018b) or ordered commercially (Banakar et al., 2019) to test the reliability of their design.

Once obtained, the guides should be assessed for editing efficiency either *in vitro* or *in vivo* prior to tissue culture and transformation. In both instances, the gRNA is incubated with their compatible Cas nuclease to form the RNP complex before testing (Woo et al., 2015; Li et al., 2016; Liang et al., 2017, 2018b; Banakar et al., 2019). The *in vitro* assay is an enzymatic reaction similar to a restriction enzyme digestion, wherein the target sequence has been amplified or cloned and subsequently incubated with the RNP complex to determine target-cleaving efficiency, while the *in vivo* assay typically relies on transfection of protoplasts with the RNP complex. *In vitro* gRNA design assays are a fast, cost-effective, and reliable method for gRNA optimization. Post-transfection, the target region of the genome is amplified and assessed enzymatically (*in vitro* assay) or sequenced [i.e., Sanger, next-generation sequencing (NGS)]], depending on resource availability and short-term goals (Woo et al., 2015). If the *in vitro* assay is performed post-transfection, the absence of cleavage is indicative of high efficiency gRNA design. Sanger sequencing addresses whether an editing event occurred, as well as the types and proportion of alleles produced. The Sanger sequence data can be analyzed by different software such as TIDE (Brinkman et al., 2018). When performed post-transfection, NGS can quantify the different edited alleles produced, and, if a reference genome is available, the relative number of off-target events.

Off-target events are unintended genetic modifications that can arise during gene-editing and are usually due to target sequence similarity (few or no mismatches) (Brazelton et al., 2015; Doench et al., 2016). The risk of off-target editing can be addressed at the gRNA design stage using various genomic strategies that typically depend on the availability of a reference genome (Manghwar et al., 2020). Without a reference genome, off-target editing sites can be unpredictable and limit the widespread application of gene-editing technology for commercial purposes. CIRCLE-seq is an *in vitro* screen for genome-wide off-target cleavage sites which has previously demonstrated potential as a genome-independent method of off-target analysis, though the technology is limited to the CRISPR-Cas9 system (Tsai et al., 2017; Lee et al., 2019). Notably, off-target effects have been observed more frequently in edited plants produced by T-DNA transformation compared to “DNA-free” RNA or RNP methods, wherein the likelihood of undesirable edits dramatically decreases as more mismatches are present between the target sequence and off-target regions (Modrzejewski et al., 2020). Therefore, introducing the CRISPR-Cas system as RNP or RNA may reduce the probability of off-target events.

### Tissue Culture

Gene editing reagents need to be introduced into plant cells using tissue culture (Figure 1C) and transformation methods (Sandhya et al., 2020). A major challenge for CRISPR-Cas genome editing in tropical crops is often the lack of efficient tissue culture and transformation protocols due to their lengthy generation times (Haque et al., 2018; van Eck, 2018). Development of efficient tissue culture and regeneration protocols will depend on the research group’s resources and the crop of interest. Thus far, there are two ways of performing tissue culture and subsequent regeneration post-transformation/transfection: chemical-based and molecular genetic-based.

The most common approach is the chemical-based strategy and is based on testing different ratios of auxin and cytokinin, two important hormones in plant development (Gaba, 2004; Chin and Tan, 2018). In this approach, plant regeneration is achieved through direct or indirect routes. The direct route involves the induction of shoots or roots directly from differentiated explant tissue, resulting in genetically stable clonal plants at a low rate of efficiency. On the other hand, the indirect route involves dedifferentiation of somatic tissue into a callus phase and subsequent production of somatic embryos occurring at a higher efficiency (Chin and Tan, 2018). Genotype-dependency, rate of somaclonal variation, and low rate of plantlet regeneration present challenges for efficient tissue culture and regeneration using this approach (Chin and Tan, 2018).

Molecular genetic-based tissue culture and transformation methods fall broadly into three categories: developmental regulators (DR), morphogenic factors, and Growth-Regulatory plus GRF-Interacting Factors (GRF-GIF). The DR-based strategy involves the expression of meristem-organizing genes, either *in vitro* or ectopically, to generate new shoots that give rise to fertile plantlets from somatic tissue (Maher et al., 2020). This method, however, is not generalizable to all crops or regulatory systems (Nasti and Voytas, 2021). On the other hand, the application of morphogenic factors is genotype-independent and generates a larger number of edited plants in less time (Lowe et al., 2016). Briefly, the overexpression of morphogenic genes, such as *Baby boom* (Boutilier et al., 2002) and *Wuschel* (Arroyo-Herrera et al., 2008), can be used to efficiently induce somatic embryogenesis directly from explant tissue without the need for a callus phase. The GRF-GIF approach uses the overexpression of specific chimeric GRF and GIF fusion proteins (Kong et al., 2020) to produce stable transformants with increased regeneration efficiency. This approach was instrumental in producing edited genotypes that were recalcitrant to previous transformation methods.

### Transformation Platform

There are two general approaches to transformation using CRISPR-Cas: transgenesis and transfection (Figure 1D). Transgenic techniques introduce exogenous DNA fragments, or transgenes, to target tissue genomes via bombardment or co-cultivation with disarmed *Agrobacterium tumefaciens* strains that insert T-DNA from binary vector systems (Bower and Birch, 1992; Fitch et al., 1993; May et al., 1995; Nasti and Voytas, 2021). The advantage of the transgene approach is that it uses selection to identify events that carry the transgene versus those that do not, facilitating the screening of CRISPR-Cas positive events. However, the integration of transgenes is non-specific and sometimes unstable (Jaganathan et al., 2018). Transfection techniques are possible because the gene editing reagents can be assembled as RNP complexes or RNA molecules *in vitro* (Zhang Y. et al., 2021) and subsequently delivered to embryogenic calli via particle bombardment or introduced in isolated protoplasts via transfection (Liang et al., 2018b). The advantage of using the transfection approach is that the edited events are transgene-free, which may or may not influence regulatory processes required for commercial release of gene edited crops. However, the disadvantage is that hundreds of events must be screened due to the absence of a selectable marker (Fister et al., 2018)*.*

### Screening

Once gene-editing reagents have been inserted into plant cells and cellular repair mechanisms have created edits, cells carrying these genetic changes need to be identified, so that regenerated plantlets that are chimeric or homozygous for the desired edit can be recovered (Figure 1E). Edited events may be phenotypically screened and subsequently verified through sequencing, or vice versa depending on the trait of interest (Kaur et al., 2018). Many methods are available (Slatko et al., 2018), but large-scale editing projects typically require a high-throughput approach. Recent advancements such as PacBio technology and Nanopore sequencing can generate long DNA (and RNA) reads at unprecedented volume, in contrast to first and second-generation sequencing which mostly produce short-read sequences (Slatko et al., 2018). The drawback of high-throughput approaches is their relatively high error rate, though the large computational capacity circumvents this problem. In the transgenic approach, selectable markers, such as antibiotic (Hardegger and Sturm, 1998) or herbicide resistance (Zhao et al., 2000), have been essential in identifying and propagating edited plants. However, concern over the presence of transgenes in the final product and the prospects of successive rounds of transformation and gene silencing have made marker elimination a more attractive approach (Veluthambi et al., 2003). Elimination of transgenes, T-DNA, and selectable markers depends on the efficiency of progeny regeneration and the ability to segregate the T-DNA from the edited allele through crosses (Russell et al., 1992; Hohn et al., 2001; Veillet et al., 2019). Furthermore, edited lines should be assessed in various field environments to ensure that the desired phenotype is heritable and stable for commercial application. Breeders must consider gene-environment interactions (GEI) and trait stability to recommend ideal varieties to growers and maximize success (Huang et al., 2016).

In the case of vegetatively propagated crops, or where traditional breeding is cumbersome or not possible, screening must be approached differently. Transgene-free methods remove the need to segregate transgenic material out of desired lines and still retain the edited event but rely on genomic analyses for selection. Briefly, polymerase chain reaction (PCR)-based and DNA sequencing-based methods are highly sensitive and specific, provided an adequate reference is available (Grohmann et al., 2019). Although limited in their application, alternative approaches include DNA hybridization assays, protein- and metabolite-based methods (Grohmann et al., 2019), and restriction enzyme assays (Kim et al., 2014; Shan et al., 2014; Vouillot et al., 2015) combined with bioinformatic analysis tools (Liang et al., 2018a). Probe-based quantitative PCR (qPCR) is a simple, robust, and rapid approach that can be applied to a broad range of genotypes (Falabella et al., 2017; Peng et al., 2018) and the use of a locked nucleic acid (LNA) probe may offer increased specificity by reducing off-target amplification (Zhang H. et al., 2021). We suggest reviewing Grohmann et al., 2019 for an overview of screening and selection methods to aid in protocol development.

## Conclusion

The promise in genome engineering technology is clear, especially in under-resourced regional tropical crops. Here, we have outlined a clear workflow to operationalize CRISPR-Cas technology in any species of interest, though it is important to understand the different cultural relevance of underutilized crops to appropriately develop resources (Gordon et al., 2021). The processes outlined here can be developed independently at different times, but all need to be in place in order to establish a CRISPR-Cas gene editing platform for improvement of your target crop.

## Data Availability

The original contributions presented in the study are included in the article/supplementary material, further inquiries can be directed to the corresponding author.
